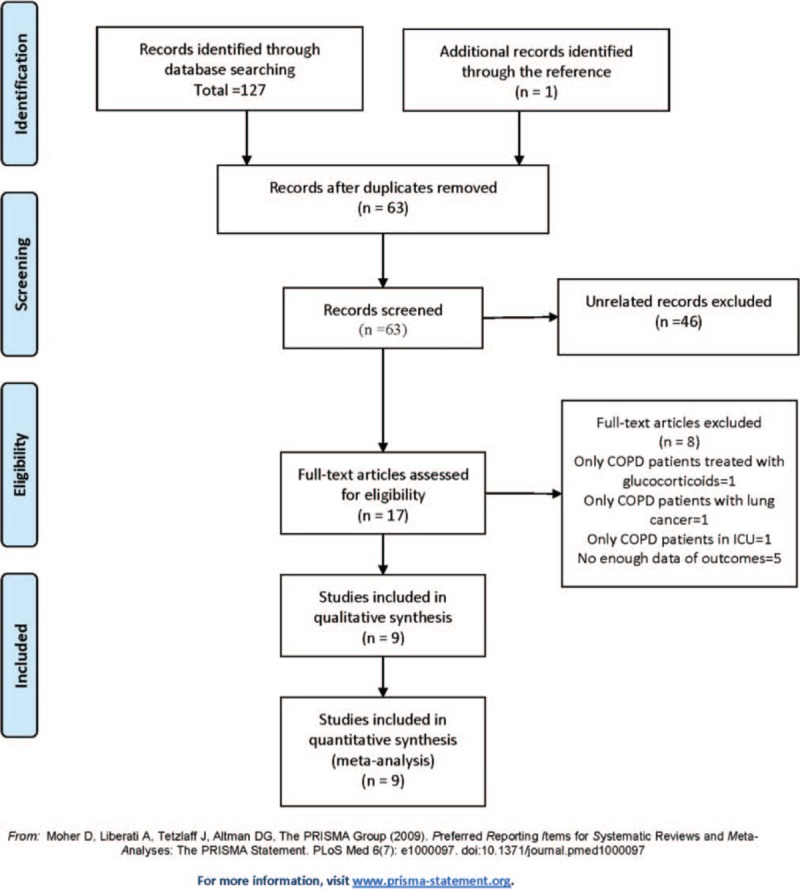# The prognostic values of neutrophil to lymphocyte ratio for outcomes in chronic obstructive pulmonary disease: Erratum

**DOI:** 10.1097/MD.0000000000016859

**Published:** 2019-08-09

In the article, “The prognostic values of neutrophil to lymphocyte ratio for outcomes in chronic obstructive pulmonary disease”,^[1]^ which appears in Volume 98, Issue 28 of *Medicine*, a few corrections need to be noted throughout the article.

In the second sentence of abstract, “conduct a meta-analysis to” should be removed.

In the Methods section of the abstract and the paper, September 2017 should be April 2019.

The number of patients in the Results section of the abstract, the first paragraph of ‘Study Characteristics’ and the first paragraph of the Discussion section should be 5384 patients.

In the results section, 8 records, not 6, were excluded. Seven studies, not 6, were rated as high quality. The references for the high quality studies should be 24,26-28,34,36.

In 3.2. Study Characteristics, 6 retrospective studies, not 5, were included in this meta-analysis.

In section 3.2.3, the number of subjects should be 4243 and the references for the 6 articles should be 25,26,28,34,35,36.

In figure 1, the last box should be ‘n = 9’, not ‘n = 8’.

**Figure:**